# Pulmonary sarcoidosis associated with psoriasis vulgaris: coincidental occurrence or causal association? Case report

**DOI:** 10.1186/1471-2466-6-26

**Published:** 2006-12-13

**Authors:** Melita Nikolopoulou, Stamatis Katsenos, Kostas Psathakis, Efstathios Rallis, Dimitrios Sampaziotis, Panagiotis Panagou, Kostas Tsintiris, Demosthenes Bouros

**Affiliations:** 1 Department of Pneumonology, Army General Hospital of Athens, Athens, Greece; 2 Department of Dermatology, Army General Hospital of Athens, Athens, Greece; 3 Department of Pathology, Army General Hospital of Athens, Athens, Greece; 4 Department of Pneumonology, Medical School, Democritus University of Thrace and University Hospital of Alexandroupolis, Alexandroupolis, Greece

## Abstract

**Background:**

Sarcoidosis is rarely associated with a distinct disease. One disease infrequently associated with sarcoidosis is psoriasis.

**Case presentation:**

This case study describes a 38-year-old male, who presented with chest pain, high-grade fever, arthralgias and a skin rash accompanied by bilateral hilar lymphadenopathy on his chest radiograph. Extensive investigations including fiber-optic bronchoscopy with bronchoalveolar lavage and labial and skin biopsies, demonstrated that two distinct clinical entities co-existed in the same patient: pulmonary sarcoidosis and psoriasis vulgaris. Combination therapy for both diseases was applied and the patient was greatly improved.

**Conclusion:**

This is the first well-documented case of sarcoidosis and psoriasis in the same patient, reported on the basis of safe and widely-used techniques that were not available until fairly recently. These disorders might share common pathogenic mechanisms that could explain their co-existence in the patient.

## Background

About 25% of sarcoidosis patients may suffer from skin lesions during the course of their disease [1]. Psoriasiform lesions have been reported in sarcoidosis [2-6]. In this paper we present a patient who showed clinical and histological features compatible with both pulmonary sarcoidosis and psoriasis vulgaris.

## Case presentation

A 38-year-old male, a heavy smoker, was admitted to our department, because of fever (38.4°C), weakness, arthralgias, non-pitting edema of the lower extremities and chest discomfort for the previous two weeks. The arthralgias were partially responsive to nonsteroidal anti-inflammatory drugs (NSAIDs). The patient also reported skin eruptions on his elbows and lower extremities that had appeared six months previously. His past medical history was unremarkable.

**Figure 1:**
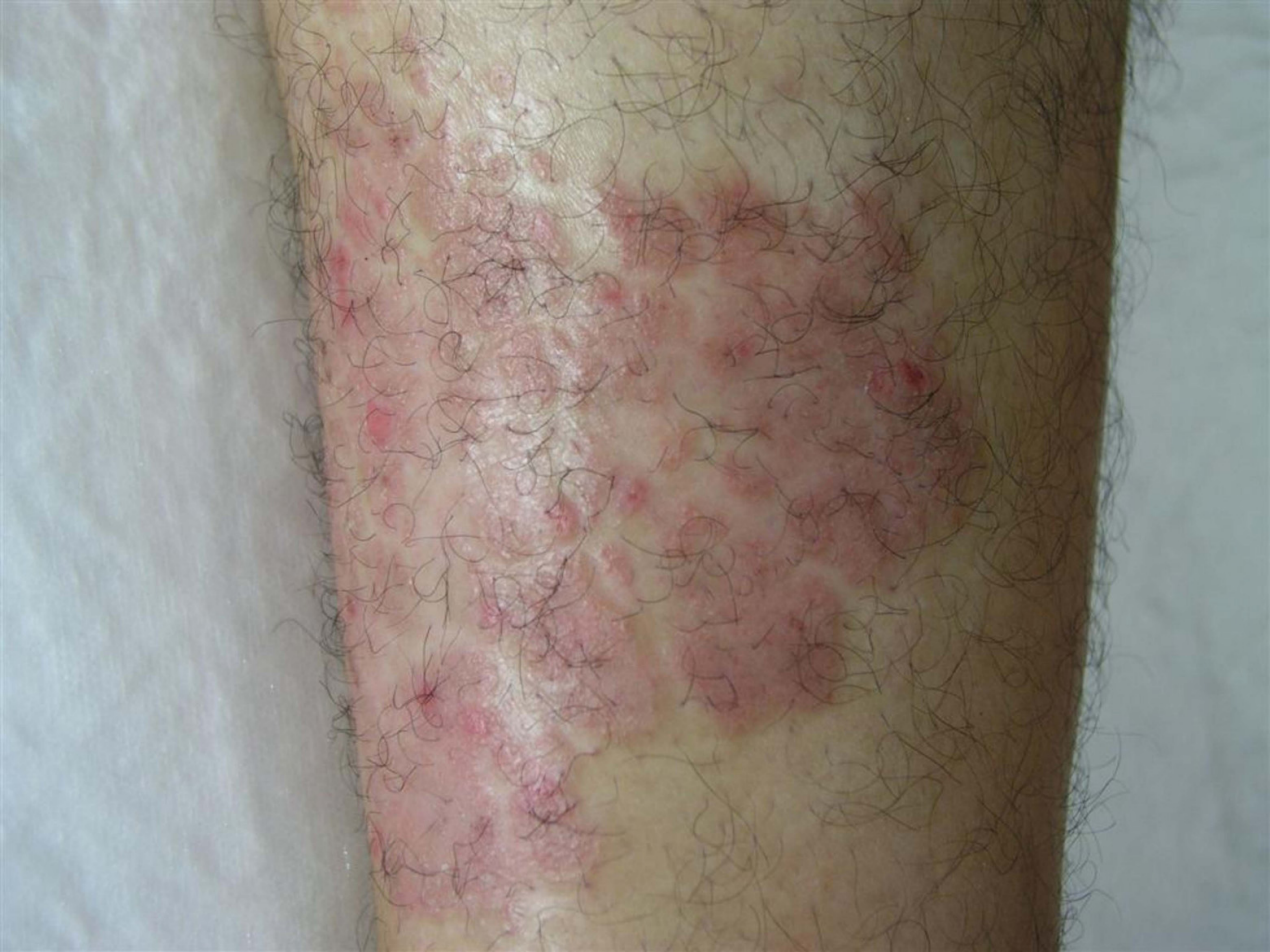
Scaly erythematous plaques of the lower extremity (psoriasis vulgaris).

On physical examination, sharply demarcated erythematous plaques covered by silvery white scales were observed on the right elbow and the lower extremities below the knees (figure 1). The patient's ankles were painful and edematous. On palpation, firm and painless subcutaneous nodules could be found on the occipital part of the scalp. The rest of the physical examination was unremarkable.

Basic laboratory tests were normal except for an increased erythrocyte sedimentation rate (57 mm/h) and C-reactive protein (45.2 mg/l). Further investigations, which included serum complement analysis, rheumatoid factor, antinuclear antibodies, antineutrophilic cytoplasmatic antibodies, immunoglobulin levels, and serological tests for hepatitis A, B and C and human immunodeficiency virus, disclosed no apparent pathologies. PPD skin test was negative. Neither arterial blood gases nor pulmonary function tests showed significant abnormalities. Radiographic examination, using x-ray and high resolution computed tomography of the chest, revealed bilateral hilar and right paratracheal lymphadenopathy with small nodules along the bronchovascular bundles. Fiber-optic bronchoscopy and bronchoalveolar lavage (BAL) were performed. BAL fluid analysis revealed lymphocytosis (35%) with an increased CD4+/CD8+ ratio (4.2). Microbiological and cytological examinations were negative. Transbronchial lung biopsies were non-diagnostic.

**Figure 2:**
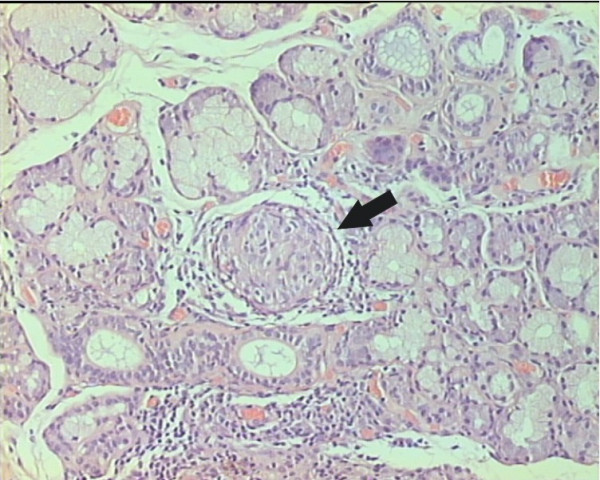
A labial biopsy reveals lobules of small salivary glands and a small, non-necrotic, sarcoid type epithelioid granuloma (arrow). (Haematoxylin & Eosin, original magnification ×100).

**Figure 3:**
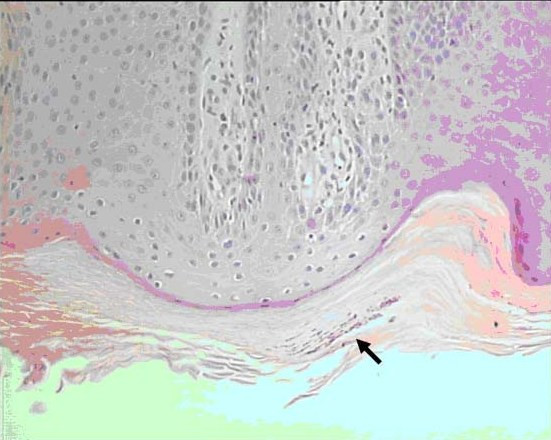
A skin biopsy reveals acanthosis of epidermis, with elongated rete ridges, thin suprapapillary epidermal plates, absent granular layer and hyperkeratosis. A Munro microabscess (arrow) is also noticed within the parakeratotic cornified layer. (Haematoxylin & Eosin, original magnification ×100).

A labial biopsy and biopsies from the cutaneous lesions were also obtained. The labial biopsy demonstrated the presence of small epithelioid (sarcoid form) granulomas (figure 2), while the skin biopsies were typical of psoriasis (figure 3). Immunohistochemical analysis of tissue samples with periodic acid-Schiff staining was negative. We concluded that the patient suffered from two separate clinical entities: pulmonary sarcoidosis and psoriasis vulgaris.

The articular manifestations responded partially to NSAIDs. Consequently, a short course of 30 mg prednisone daily and topical therapy to manage the psoriatic lesions were applied. This resulted in a significant clinical improvement. Three months after his discharge the patient remains asymptomatic.

## Discussion

Sarcoidosis is predominantly seen in young and middle-aged patients and may involve multiple organs [7]. Although the cause of sarcoidosis remains uncertain, much is known about the immunology of this disease. The immunological hallmark is an accumulation of activated CD4+ T lymphocytes at the sites of inflammation. Interaction between the CD4+ cells and alveolar macrophages leads to a Th1-skewed cytokine profile that orchestrates the ensuing granulomatous inflammatory process [8].

The diagnosis of sarcoidosis is commonly established on the basis of compatible clinical and/or radiological features, supported by the histological findings of noncaseating granulomas and the exclusion of other diseases with a similar clinical or histological picture [7]. The recommended diagnostic procedure is fiber-optic bronchoscopy combined with various biopsy techniques (endobronchial biopsy, transbronchial lung biopsy, transbronchial needle aspiration and biopsy) as well as BAL. Studies of the lymphocyte subpopulations in the BAL fluid may be diagnostically important. A CD4+/CD8+ ratio of >3.5 in a patient with a typical clinical and radiological picture is very specific for sarcoidosis and may obviate the need for further invasive diagnostic procedures [8]. If bronchoscopy fails to confirm the diagnosis, labial biopsy is a fairly useful method for providing histological confirmation of the disease [9]. In the case presented here, the compatible radiographic findings, the results of BAL fluid analysis and the characteristic histological findings from the lip biopsy were considered sufficient to support the diagnosis of sarcoidosis.

Psoriasis is a common inflammatory skin disorder characterized by erythematous, sharply demarcated papules and rounded plaques, covered by silvery micaceous scales which most commonly appear on the elbows, knees, scalp, umbilicus and lumbar area [10]. Skin biopsy may show a variable histological picture but is usually diagnostic in early lesions or at the advancing edge of a well-established plaque. The characteristic histological features in psoriasis include proliferation of epidermal keratinocytes and hyperkeratosis as well as infiltration of immunocytes along with angiogenesis, and subsequent typical thickening and scaling of the erythematous skin. More specifically, the epidermis in psoriatic skin is characterized by dramatic pathological alterations, such as profound acanthosis with elongation of epidermal rete ridges. Likewise, neutrophils migrate through the epidermis and form Munro microabscesses beneath the stratum corneum; this is one of the most salient features of the disease [10]. The findings from skin lesions in our patient were identical to those mentioned above.

Compelling circumstantial and experimental evidence suggests that the primary immunopathogenesis of psoriasis is T-lymphocyte based, so least in part it resembles sarcoidosis. The immigrant immunocytes interact with resident epithelial and mesenchymal cells to generate the psoriatic lesion. Once activated, the T-lymphocytes excrete a panel of Th1-type proinflammatory cytokines, which are thought to explain many of the histopathological changes seen in psoriatic skin. It is likely that the inflammation of psoriasis is a balance between the adaptive (T-cell mediated) immune response and innate immune responses [11].

Sarcoidosis is rarely associated with other diseases, and most of these are characterized by a Th1/Th2 imbalance (common variable immunodeficiency, autoimmune diseases etc.) [12]. Moreover, the skin manifestations in sarcoidosis are not always granulomatous (e.g. erythema nodosum) and may result from a systemic immunological reaction. Such a reaction might explain the presence of psoriatic lesions in a patient with sarcoidosis. Thus the coexistence of sarcoidosis and psoriasis might not be coincidental. Both diseases share common pathogenic pathways and the occurrence of psoriasis in a sarcoidosis patient might result from a variable response to a common antigenic stimulus. Enhancement of the Th1 immune response is well recognized in both diseases. Moreover, the promising results obtained in both sarcoidosis and psoriasis patients after application of anti-TNF alpha agents (e.g. infliximab, adalimumab, etanercept) further support this hypothesis, since these therapeutic agents may alter pathogenic mechanisms common to both disorders [11,13-15]. It has also been reported that the expression of pso p27, a psoriatic scale antigen linked to the pathogenesis of psoriasis, is markedly increased in the lungs of patients with pulmonary sarcoidosis [16].

## Conclusion

The number of patients reported with this combination of diseases (sarcoidosis and psoriasis) is small and more data are necessary to clarify the relationship. This is the first reported case of pulmonary sarcoidosis and psoriasis diagnosed by simple, safe and novel routine procedures. The coexistence of these disorders supports the hypothesis that they share common pathogenic mechanisms.

## Abbreviations

NSAIDs: non-steroidal anti-inflammatory drugs, PPD: purified protein derivative, BAL: bronchoalveolar lavage.

## Competing interests

The author(s) declare that they have no competing interests.

## Authors' contributions

MN was responsible for patient management and prepared the manuscript. SK and KP performed the fiber-optic bronchoscopy and the various biopsy techniques (mucosal and transbronchial lung biopsy, transbronchial needle aspiration and biopsy) and bronchoalveolar lavage (BAL), and participated in patient follow-up. PP and KT were involved in observing the patient and helped to draft the manuscript. ER carried out the labial and skin biopsies. DS performed the pathology tests. DB critically revised the manuscript and approved the final version. All authors read and approved the final manuscript.

## Pre-publication history

The pre-publication history for this paper can be accessed here:
